# Navigation Services to Avoid Rehospitalization among Medical/Surgical Patients with Comorbid Substance Use Disorder: Rationale and Design of a Randomized Controlled Trial

**DOI:** 10.20900/jpbs.20200013

**Published:** 2020-06-12

**Authors:** Courtney D. Nordeck, Christopher Welsh, Robert P. Schwartz, Shannon G. Mitchell, Kevin E. O’Grady, Laura Dunlap, Gary Zarkin, Stephen Orme, Jan Gryczynski

**Affiliations:** 1 Friends Research Institute, Baltimore, MD 21201, USA; 2 University of Maryland Medical Center, Baltimore, MD 21201, USA; 3 Department of Psychology, University of Maryland, College Park, College Park, MD 20742, USA; 4 RTI International, Research Triangle Park, NC 27709, USA

**Keywords:** patient navigation, substance use disorders, hospitalization, emergency department visits, health service utilization, addiction consultation

## Abstract

Substance use disorders (SUDs) are associated with significant morbidity and mortality and contribute to inefficient use of healthcare services. Hospitalized medical/surgical patients with comorbid SUD are at elevated risk of hospital readmission and poor outcomes. Thus, effective interventions are needed to help such patients during hospitalization and post-discharge. This article reports the rationale, methodological design, and progress to date on a randomized trial comparing the effectiveness of Navigation Services to Avoid Rehospitalization (NavSTAR) vs Treatmentas-Usual (TAU) for hospital medical/surgical patients with comorbid SUD (*N* = 400). Applying Andersen’s theoretical model of health service utilization, NavSTAR employed Patient Navigation and motivational interventions to promote entry into SUD treatment, facilitate adherence to recommendations for medical follow-up and self-care, address basic needs, and prevent the recurrent use of hospital services. As part of the NavSTAR service model, Patient Navigators embedded within the SUD consultation service at a large urban hospital delivered patient-centered, proactive navigation and motivational services initiated during the hospital stay and continued for up to 3 months post-discharge. Participants randomized to TAU received usual care from the hospital and the SUD consultation service, which included referral to SUD treatment but no continued contact post-hospital discharge. Hospital service utilization will be determined via review of electronic health records and the regional Health Information Exchange. Participants were assessed at baseline and again at 3-, 6-, and 12-month follow-up on various measures of healthcare utilization, substance use, and functioning. The primary outcome of interest is time-to-rehospitalization through 12 months. In addition, a range of secondary outcomes spanning the medical and SUD service areas will be assessed. The study will include a health economic evaluation of NavSTAR. If NavSTAR proves to be effective and cost-effective in this high-risk patient group, it would have important implications for addressing the needs of hospital patients with comorbid SUD, designing hospital discharge planning services, informing cost containment initiatives, and improving public health.

## INTRODUCTION

Substance use disorders (SUDs) are strongly associated with repeat hospital admissions and emergency department (ED) visits [1–8]. Individuals with SUDs often require hospital re-admission for various reasons, such as lack of adherence to post-discharge treatment plans or medication regiments, prioritization of substance use over self-care, and difficulty navigating systems of care [5,9,10]. Given the increased utilization of healthcare services and associated costs, there is considerable public health and economic interest in developing cost-effective approaches to engage individuals with comorbid medical conditions and SUDs in appropriate outpatient medical care and SUD treatment.

In recent years, there has been a growing focus on preventing high levels of hospital utilization and readmissions [11]. The Center of Medicaid and Medicare Services (CMS) implemented a Hospital Readmission Reduction Program (HRRP) which levies reimbursement penalties on hospitals with high 30-day readmissions for certain diseases [12]. These policies have generated an interest in identifying approaches that foster a smooth continuity of care from the hospital to community care services that could then reduce avoidable readmissions. Extant research suggests that interventions that contain more components, involve more individuals in care delivery, and support patient capacity for self-care were more effective in reducing hospital readmission [13]. However, there is a substantial amount of heterogeneity among such interventions [14]. Additionally, most existing interventions target populations with specific diseases, such as chronic heart failure. There has been less research available on interventions to reduce hospital admissions targeted for individuals with comorbid medical conditions and SUDs.

### Promising Interventions for Hospitalized Patients with SUD

#### Patient navigation

Patient Navigation (PN) is a form of strength-based case management that strategically guides individuals through the complexities of the existing healthcare system by identifying barriers to treatment entry and adherence to healthcare, in order to alleviate poor outcomes and reduce health disparities. The technique is patient-centered and provides tailored one-on-one assistance to address barriers to facilitate service utilization in the community. Extant research has found that PN can improve cancer screening and follow-up rates [15], as well as entry, adherence, and viral load outcomes in HIV treatment [16,17]. Randomized controlled trials (RCTs) have found that similar approaches, including outreach case management, can increase rates of SUD treatment entry [18–23]. However, not all studies of PN have found it to be effective. One large trial found that PN did not have an impact on viral suppression among hospital patients with HIV and substance use at 12-month follow-up [24].

PN can take various forms, with different levels of staffing and professional competencies of the Navigators. The broad umbrella of PN services encompasses a wide range of staff training and experience, from peers in recovery with very basic training to Master’s-level social workers who can deliver skilled therapeutic interventions and interact directly with medical staff as part of a patient’s care team. While different staffing models may have their potential advantages and disadvantages, the latter model of PN utilizing clinically trained Master’s level social workers was used in the NavSTAR trial.

#### Motivational interventions

Motivational interventions offer promising results in helping individuals reduce alcohol or drug use. A Cochrane Review of 59 RCTs concluded that motivational interventions are superior to no-treatment controls in facilitating short- and medium-term reductions in substance use [25]. Additionally, some studies show that motivational interventions can improve rates of SUD treatment entry in treatment-seeking and non-treatment seeking populations and are associated with a reduction in hospital admissions [18,19,26–28].

### Coupling Patient Navigation and Motivational Interventions

While there have been mixed results of the efficacy of PN and related approaches in different care settings, it is reasonable to assume that coupling patient navigation and motivational interventions might be useful to assist hospitalized patients in maintaining continuity of care and promoting basic needs while transitioning back to the community from the hospital. Research and field experience point to two major categories of barriers to appropriate medical and SUD treatment service utilization for individuals with SUDs: (1) *internal* factors, many of which are amplified by SUD pathology (e.g., low problem recognition, ambivalence, fluctuating motivation, disorganization), and (2) *external* constraints (e.g., transportation, health insurance, requirements for admission or subsidized treatment such as ID cards and proof of residence, unclear processes in fragmented systems of care). Engagement in appropriate treatment services requires simultaneous resolution of both the internal and external barriers that may hamper such engagement.

We posited that coupling Patient Navigation with motivational interventions could help to resolve these barriers. This approach, which we term Navigation Services to Avoid Rehospitalization (NavSTAR), would enhance the likelihood that discharged hospital patients with co-occurring SUD engage in recommend follow-up medical treatment and self-care as well as SUD treatment, thereby supporting medical stabilization and reducing risk of rehospitalization. It would leverage hospitalization as a “reachable moment” to increase motivation to reduce substance use and engage with appropriate outpatient services [29]. Even a small but reliable reduction in rehospitalization rates for this population could offset the costs of the intervention and yield substantive public health benefits.

### Conceptual Model

**Figure 1.**
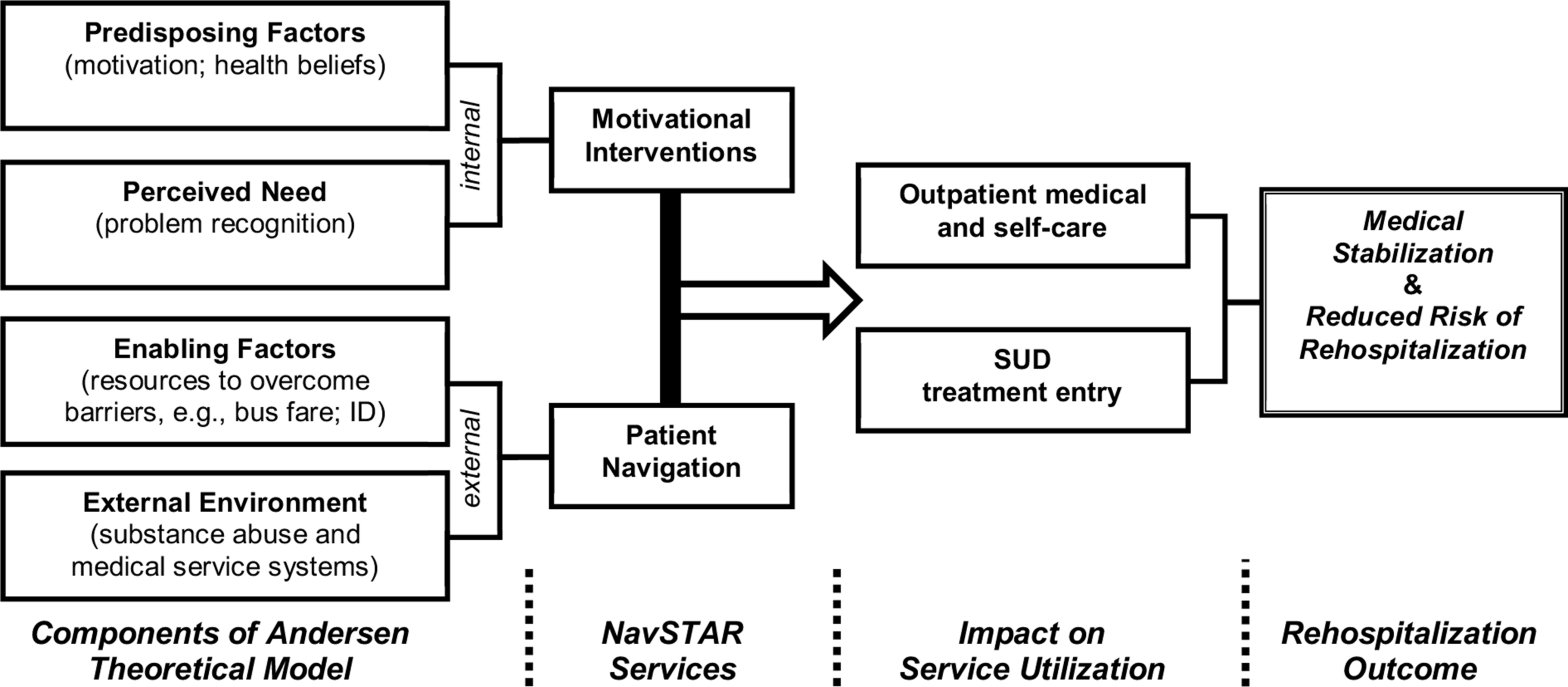
Andersen model of health service utilization applied to NavSTAR.

Figure 1 shows Andersen’s conceptual model of health services utilization adapted to the current study [30,31]. This model specifies key determinants of health service utilization that align directly to the *internal* and *external* barriers experienced by many individuals with SUDs. These determinants include *predisposing factors* (e.g., motivation and health beliefs), *perceived need*, and *enabling factors* (e.g., access to resources and services), which impact how the individual will deal with the constraints of the *external environment* (e.g., outpatient medical and substance abuse services).

NavSTAR targeted services to each of these areas. Patient Navigators delivered motivational interventions designed to increase patients’ willingness to engage in appropriate outpatient services by targeting the predisposing factors of individual motivation and health beliefs. Such interventions can also impact perceived need by prompting patients to consider the negative consequences of substance use.

Once they catalyzed patients’ readiness for taking action, Navigators followed through to systematically address external barriers to engaging in appropriate services. They targeted enabling factors by assisting patients with necessary resources (e.g., transportation, insurance) and services (e.g., via reminders and meeting patients at their appointments). Navigators also assisted patients to traverse the service systems in the external environment by helping them to overcome the bureaucratic obstacles to engaging in care (e.g., through advocacy for patients with providers). In addressing this range of barriers, NavSTAR aimed to increase the likelihood of entry into outpatient medical care and substance abuse treatment, which in turn supported medical stabilization and reduced risk of subsequent rehospitalization.

The purpose of the NavSTAR study was to examine the effectiveness and cost-effectiveness of Navigation Services to Avoid Rehospitalization (NavSTAR) *vs*. treatment-as-usual (TAU) for medically ill hospital patients with comorbid SUDs recruited from a large urban academic medical center.

## METHODS

### IRB Approvals and Data and Safety Monitoring

The FRI and University of Maryland (UMD) Institutional Review Boards (IRB) approved the study. The study was registered at ClinicalTrials.gov (NCT02599818). A federal Certificate of Confidentiality was obtained to protect the confidentiality of participants’ data.

The study was monitored by a Data and Safety Monitoring Board (DSMB). The FRI IRB, UMD IRB, DSMB members, and NIDA (the study sponsor) monitored recruitment, retention, and study safety. All Serious Adverse Events were reported to the IRBs, DSMB, and NIDA medical monitor, as necessary.

### Study Design

This study was a parallel two-group randomized controlled trial that examined NavSTAR compared to TAU. Participants were 400 hospitalized patients who met diagnostic criteria for opioid, cocaine, and/or alcohol use disorder at University of Maryland Medical Center in Baltimore City.

### Study Aims and Hypotheses

The primary research aims were to determine the effectiveness of NavSTAR compared to TAU in regards to (1) Medical Services Outcomes (e.g., hospital inpatient and emergency department utilization), (2) Substance Use Services and Related Outcomes (e.g., SUD treatment entry; substance use; SUD diagnostic criteria; risk behaviors) and (3) Economic Outcomes (e.g., cost-effectiveness). We hypothesized that NavSTAR will be superior to TAU in each of these domains.

### Study Site

The study was conducted by Friends Research Institute (FRI) in Baltimore City. The recruitment was conducted at the University of Maryland Medical Center (UMMC), a major urban medical center in downtown Baltimore.

UMMC has an addiction consultation liaison (CL) service that has been in continuous operation for many years [32,33]. This model of addiction consultation has become increasingly used in hospital settings to address the needs of hospital patients with SUDs and support linkage to treatment [34–37]. The UMMC CL service responds to requests from the Internal Medicine and Surgery/Trauma departments regarding patients whose substance use history may affect their medical prognosis [32,33]. The service consists of a multidisciplinary team, which includes a psychiatric doctor, part-time addiction-boarded psychiatrists, nurses, a licensed addiction counselor, a licensed social worker, and medical/psychiatric residents and addiction medicine fellows. This team regularly conducts assessments at the bedside and provides a number of substance use focused services, such as motivational counseling and initiation of medications to treat opioid use disorder and alleviate withdrawal.

### Inclusion/Exclusion Criteria

Inclusion criteria were: (1) age 18 of older; (2) current DSM-5 criteria for alcohol, cocaine, and/or opioid use disorder (at the time of screening); and (3) willing and able to provide informed consent in English.

Exclusion criteria were: (1) enrollment in SUD treatment 30 days prior to hospitalization; (2) residency outside of Baltimore city; (3) pregnancy; (4) planned discharge to a long-term inpatient care facility (e.g., hospice); or (5) hospitalization for a suicide attempt.

### Participants

**Figure 2.**
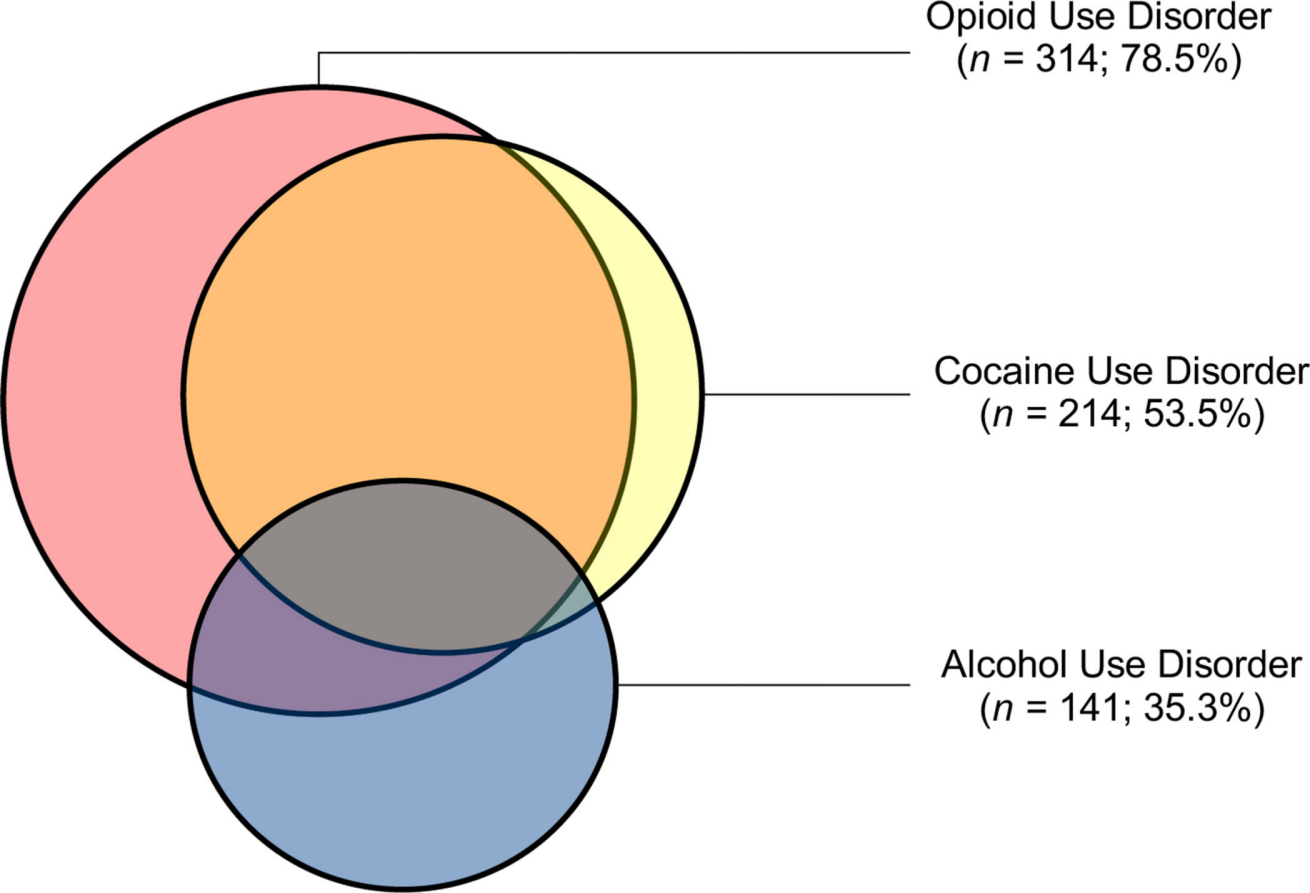
Substance use disorder criteria breakdown of NavSTAR participants.

A total of 400 participants were enrolled into the study from March 2016 through May 2018. The sample was primarily male (57%) and Black race (55.5%), with a mean age of 45.1 years (SD = 12.3). The majority of the sample met diagnostic criteria for opioid use disorder (78.5%), while over half met criteria for cocaine use disorder (53.5%) and approximately a third met criteria for alcohol use disorder (35.3%). Over half of the sample met criteria for more than one substance use disorder (58.3%; see Figure 2). Participants also reported moderately high rates of unemployment (42.3%), disability (35.0%), and homelessness (43.0%).

### Recruitment

Staff members of the addiction consultation liaison team referred medically stable adults who expressed preliminary interest to speak with the Research Assistant (RA) about the study. The RA met with the patient privately to discuss the study and to screen them for eligibility.

### Informed Consent

During the research visit, the RA described the study, reviewed the informed consent form, and reviewed the risks and benefits of participation. The RA emphasized that participation was completely voluntary and would not impact the treatment they would regularly receive from the hospital. To verify that the patient understood the purpose and procedures of the study (and thus was able to provide informed consent), the RA administered a brief 6-item consent quiz on which individuals must have received a perfect score within three attempts to be deemed eligible.

### Screening, Randomization, and Follow-up Procedures

The RA completed the eligibility checklist to verify that the patient was eligible for participation prior to enrollment and then proceeded with the informed consent process for those individuals who met all other eligibility criteria. After the individual provided written informed consent, the RA administered the baseline instruments.

Following the completion of all baseline measurements, the RA opened the next randomization envelope and informed the participant of his/her assigned study condition. Participants were assigned to conditions using a random permutation procedure, so that, for each block of 2, 4, or 6 participants, one half would be assigned at random to the NavSTAR condition, and one half to the TAU condition. The Project Manager provided the RA with sealed opaque envelopes based on the randomization sequence. Participants received compensation following the completion of all baseline procedures (including randomization), in addition to receiving compensation ($40) for completion of each of the three follow-up assessments.

### Data Management

RAs completed baseline assessments on paper forms and then entered the data directly into the University of Pennsylvania’s Data Management Unit (DMU), a web-based data entry system. Paper forms were necessary because the RAs did not have reliable internet connection in the hospital. Data from follow-up interviews were directly entered by the RA using the DMU.

### Study Conditions

#### Treatment-as-usual (TAU)

Individuals assigned to the TAU condition received standard care from the hospital as appropriate. The CL service continued to work with TAU participants as usual, providing resources and recommendations such as referrals to community-based treatment, education about drug and alcohol use, and acute withdrawal management. If medically appropriate, the CL service also facilitated initiation of medications for opioid use disorder (methadone or buprenorphine), and provided referrals for continued treatment post-discharge. All other services that were normally provided by hospital care teams were available to participants.

#### Patient navigation services (NavSTAR)

Individuals assigned to the NavSTAR condition received the same standard care from the CL service and other hospital staff as described for TAU. In addition, NavSTAR participants were seen by one of the study’s Patient Navigators at the bedside during their initial hospitalization. During the initial meeting, the Patient Navigator conducted an assessment of readiness for SUD treatment and behavior change, medical needs, and social determinants of health. The Patient Navigator delivered a motivational intervention as appropriate, developed rapport, and made a post-discharge plan with the participant. The Patient Navigator was available to work with the participant for up to three months post-discharge. During this time, based on a patient navigation manual developed for the project that built upon extant research [23,38], the Patient Navigator met with the patient at the hospital bedside to discuss patient-specific barriers, review the recommendations of the medical team (including the CL team), and to initiate rapport-building.

Using patient navigation services and motivational intervention techniques, the Patient Navigator continued to coordinate with the patient following discharge for up to 3 months to identify available resources and strategies to resolve discussed barriers (e.g., transportation, insurance coverage, governmental assistance). Navigators had a small fund available (used on a case-by-case basis and typically not exceeding $200 total per participant) to assist patients in obtaining ID cards, transportation, low-cost phones/cell phone minutes, and other related items. Table 1 shows examples of common barriers and how the Navigators would address them.

Prior to the start of the study, the Patient Navigators integrated themselves into the well-established CL team within the hospital. The Navigators became well-versed in the treatment modalities and providers in the local SUD and medical systems by visiting various clinics and establishing communication with intake coordinators and clinical teams in the surrounding area. For example, Navigators created a database of community-based programs with details on requirements of each program in order to inform their referral process.

### Assessments

Assessments were conducted by well-trained RAs as outlined in Table 2. The RAs were blinded to study condition at baseline (assessment was conducted prior to random assignment). RAs were not blind to assignment at follow-up visits. All participants were sought for follow-up assessments at 3-, 6-, and 12-months post-discharge, as available (i.e., alive, not incarcerated). Assessments consisted of a battery of instruments to measure outcomes including, by not limited to: substance use (Addiction Severity Index-Lite) [39], SUD severity (modified World Mental Health Composite International Diagnostic Interview [WMH-CIDI]), risk behaviors (Risk Assessment Battery) [40], health and functioning (World Health Organization Quality of Life [WHOQOL-BREF]) [41], psychological stress (Kessler-6 scale) [42], healthcare utilization, and economic cost data. Urine drug screenings were also collected at 3-, 6-, and 12-month follow-up interviews and tested by an approved rapid drug screen card for opiates, oxycodone, methadone, buprenorphine, cocaine, marijuana, amphetamines, and benzodiazepines.

### Outcomes

The primary outcome was pre-specified as time-to-rehospitalization over a 12-month time frame. A key secondary outcome was inpatient rehospitalization within 30 days. Additional outcomes included the time-to-event, number, and rate of hospital utilization events (including inpatient hospitalizations and emergency department utilization events and cumulative days), all-cause mortality, addiction treatment entry, use of other healthcare services, and patient-reported quality of life. Opioid, cocaine, and alcohol use were examined at each time point by self-report as well as urine test biomarkers for opioids and cocaine. DSM-5 criteria for opioids, cocaine, and alcohol use disorder was examined for the past 30 days at each time point.

A multi-pronged approach was used to ascertain hospital service utilization. The initial plan was to obtain records for hospitalization events that were disclosed by participants during follow-up interviews, but a more objective and comprehensive strategy was ultimately used, as detailed below.

#### Self-report and record verification

Retrospective self-reported information on inpatient and ED utilization data were collected at 3-, 6-, and 12-month follow-up interviews. Using timeline follow-back techniques, RAs inquired whether participants were hospitalized or visited an ED since their last interview. Utilizing a calendar containing relevant anchor dates (such as holidays, special events), RAs probed participants about specific admission and discharge dates, reasons for admission, and the hospital in which the encounter occurred. If the participant reported a hospitalization or ED visit, the RA then asked the participant to sign the appropriate release of information and the medical records for the reported event were requested from each hospital. Data were abstracted from the medical record and recorded into a secured, de-identified database. In addition to self-report, information on hospital service utilization at the index hospital and affiliated institutions was abstracted directly from the hospital’s electronic health record.

#### Health information exchange

The NavSTAR project became the first randomized controlled trial approved to access the regional health information exchange (HIE) for research purposes [43]. The HIE, known as the Chesapeake Regional Information System for our Patients (CRISP), serves Maryland and Washington DC and exchanges clinical and healthcare encounter data across all hospitals in the area, offering a far more comprehensive and reliable source of information on hospital service utilization than self-report. Data on hospitalizations and ED visits were abstracted from CRISP for the 12-month study period and recorded into the hospitalization database. Outcomes related to hospital service utilization (inpatient admissions, emergency department visits) were based on objective records from the CRISP HIE, the index hospital’s EHR, and records received from other hospitals.

#### Division of vital records

Verified death data were collected from the state Division of Vital Records for any participant who died during their 12-month follow-up period in the study. Death certificate records, which included date and cause of death, were completed by the Office of the Chief Medical Examiner. These records were requested by research staff if they had been informed of a participant’s death from a reliable proxy (e.g., family or friend), or if an event of death had been noted in the health service utilization records. In addition, death record searches were requested for participants who were lost-to-follow-up at 12 months and did not have evidence of life in other databases.

### Sample Size and Power

The study originally targeted recruitment of 420 participants. However, due to various administrative issues, there was a delay in the initial launch of the study, as well as factors during the course of the study (e.g., staff turnover) that periodically delayed recruitment yield. In order to ensure adequate resources for completing 12-month follow-ups, recruitment was closed with *N* = 400 enrolled participants. This had minimal impact on statistical power, with power to detect the targeted effect size decreasing from 0.84 to 0.82. The description of statistical power, below, is provided for the actual sample size of 400.

Power for the primary outcome of time-to-rehospitalization was calculated using the -stpower- module for Cox regression in Stata 12. Power was calculated to detect a hazard ratio of 1.5 at a Type I error rate of 0.05 (two-tailed), under a range of assumptions regarding the 12-month rehospitalization rate. Under the conservative assumption of a 50% event rate, *N* = 400 yielded power of 0.82 to detect a hazard ratio of 1.5 between study conditions. These calculations provided a rough estimate, as the actual event rate was considerably higher and participants commonly experienced multiple events over the 12-month observation period, necessitating the use of modeling extensions that allow for multiple events.

### Statistical Analysis

The primary outcome was defined as days-to-hospitalization because it offers the most comprehensive view of rehospitalization within the parameters of the study, while having good statistical power. Rates of rehospitalization within 30 days were examined as a secondary outcome because of its centrality in current health policy, including but not limited to Medicare’s reimbursement penalties [14,44,45]. Entry into medical care and SUD treatment are included as secondary outcomes because they represent conceptual underpinnings of the intervention. Urine screen tests results were used as an objective measure of drug use, while diagnostic criteria were used to determine SUD remission and clinically significant impairment related to substance use. Both measures are widely used outcomes in studies of SUD treatment RCTs. Finally, we included HIV-risk behaviors because individuals with SUD are at disproportionately high risk of engaging in behaviors that place them at risk for HIV and reducing such behaviors would have important public health benefits.

The primary outcome of days-to-rehospitalization through 12 months are examined using semi-parametric Cox proportional hazards regression [46], a useful method for analyzing time-to-event phenomena because it uses all of the data and accommodates censoring. It makes no assumptions about the distribution of event times and can handle multiple event data with minor adjustments [47].

Secondary outcomes are examined using a generalized linear modeling framework, with appropriate extension to mixed modeling for outcomes measured repeatedly, for which we examine change from baseline (i.e., Quality of Life and HIV risks) or across follow-up points. Generalized linear (mixed) modeling was chosen as the statistical method because it allows for a wide range of distributional assumptions of the outcomes and permits consideration of endpoint outcomes as well as change over the 12-month assessment period [48].

### Economic Analysis

The economic analysis estimated the cost and cost-effectiveness of the NavSTAR intervention. Cost estimates were calculated following an activity-based approach which produces the cost of each patient’s intervention and healthcare costs [49,50]. The total intervention cost per patient of the NavSTAR arm was the sum of (1) staff labor costs (e.g., the value of time spent providing intervention services and related administrative activities), (2) costs of building space and vehicles used in the intervention and administrative activities, and (3) costs of any other intervention supplies, support funds, or materials. These data were collected through staff time dairies and an economic interview with the study team to identify wages and the other cost components.

Healthcare costs were calculated by multiplying healthcare utilization by unit costs and summed to calculated total healthcare costs per patient. Outpatient healthcare utilization, including substance use disorder treatment, will be tracked using a modified version of the Economic Form 90 (EF90) [51]. Days in hospital and emergency department visits were obtained from the CRISP HIE. The unit costs for monetizing healthcare use were drawn from the literature and public data sources [52].

Our cost-effectiveness analysis includes intervention costs and healthcare costs calculated from a provider (e.g., hospital, outpatient clinic) perspective. We expect to conduct cost-effectiveness analyses for the outcome of the number of rehospitalization events and possibly for selected secondary outcomes. For each outcome measure, we calculate incremental cost effectiveness ratios (ICERs) by dividing the incremental cost of NavSTAR relative to TAU by the incremental difference in the desired outcome between NavSTAR and TAU. The ICER shows the cost of an additional unit of the desired outcome, for example, the cost incurred to achieve reduced rehospitalization.

To understand the uncertainty of our ICER estimates, we plan to use bootstrapping methods to calculate cost-effectiveness acceptability curves (CEACs) which show the joint probability of our cost and outcome estimates [53,54]. We also conduct sensitivity analyses to assess whether the economic results are affected by assumptions made in estimating costs and modeling outcome measures. One-way sensitivity analyses are utilized to examine the effect of changing one of the economic parameters holding all other parameters constant.

## PROGRESS TO DATE AND FUTURE DIRECTIONS

### Completion of Recruitment

As noted above, the study closed recruitment with 400 participants enrolled, 200 of which were randomized to TAU and 200 of which were randomized to NavSTAR.

### Monitoring of Service Delivery

All except one NavSTAR participants received the allocated intervention, defined as having at least one service encounter with their Navigator. The one participant who did not meet with a Navigator left the hospital against medical advice and could not be subsequently located in the community. All of the remaining participants had at least one meeting with their Navigator, although there was considerable variability in the number and duration of service encounters. Patient Navigators tracked “clinically meaningful” service encounters in a project database, noting the type (e.g., in-person, phone, etc.), purpose, and duration of the encounter, as well as whether the encounter included motivational intervention. During the course of the project, weekly multidisciplinary team meetings were held to debrief about cases and progress, monitor and reinforce fidelity to the principles of the intervention, and offer constructive feedback to the Patient Navigators. As a check against contamination, Navigators confirmed at each of these meetings that they had no contact with TAU participants.

### Abstraction of HIE Data

Data on hospitalizations were abstracted from the CRISP HIE for the 12-month period post-discharge from the index hospitalization for all participants. This covered hospital encounters across the entire state of Maryland, Washington DC, and the surrounding region. Where applicable, HIE data were cross-checked with electronic health records from the University of Maryland Medical Center and affiliated institutions. An initial comparison of HIE data and self-report, which confirmed the superiority of HIE data in ascertaining hospital service utilization, is reported elsewhere [43].

### Anticipated vs Actual Loss-to-Follow-up

The experience with obtaining follow-up data in this study was mixed. On one hand, the ability to access the CRISP HIE effectively provided comprehensive data on the entire sample where it mattered most: the key hospital service utilization outcomes of interest [43]. On the other hand, for secondary outcomes relying on participant self-report and urine testing, there was considerable loss-to-follow-up that exceeded what was initially anticipated. This circumstance was largely due to the characteristics of this population, which made participants exceptionally difficult to locate. For example, over 40% of participants were homeless, with more unstably housed or in transient living situations. Thus, overall rates of follow-up interview completion for the full sample of 400 at 3-, 6-, and 12-months were 63.0%, 61.25%, and 56.0%, respectively. However, many of the participants who were not interviewed were not available for follow-up due to death, medical instability, or incarceration. Among participants who were available for interview, follow-up rates were somewhat better (69.4%, 71.6%, and 68.1% at 3-, 6-, and 12-months, respectively), albeit still lower than anticipated. Hence, the study has complete objective data on hospital service utilization outcomes but has missing data on outcomes gathered via in-person follow-up visits. There were no significant differences in rates of follow-up between the treatment groups (*p*_s_ > 0.05 at each follow-up point).

### Future Directions

Future plans include analysis and reporting of outcome data, focusing on hospital service utilization over the 12-month period post-discharge from the index hospitalization. Additional analyses and reports are planned that focus on outcomes related to SUD treatment entry and drug use outcomes. Data analyses will also be conducted to examine the impact of the intervention from a health economics perspective. Analyses focused on specific subpopulation characteristics (e.g., gender, homelessness) will also be considered. Additional secondary analyses using mixed quantitative and qualitative interview data will seek to better understand the experiences of hospital patients with SUD who are discharged to acute and sub-acute skilled nursing facilities. The NavSTAR trial is poised to answer many important questions about how to address the complex needs of hospital patients with comorbid SUDs.

## Figures and Tables

**Table 1.** Examples of potential barriers to engaging in care and navigator response.

Example of Barrier	Navigator response
Ambivalence for medical and/or SUD treatment	Use motivational interventions to explore and resolve ambivalence; Deliver basic education to address health beliefs and increase health literacy about treatment options.
Discomfort interacting with treatment staff	Explore underlying reasons for patient discomfort (e.g., low literacy; perceived stigma) and address with education and/or role playing; Advocate for patient with treatment staff.
Lacks health insurance or has insufficient coverage	Identify appropriate insurance eligibility and options; Help patient fill out application and interface with insurance bureaucracies on patient’s behalf.
Cannot afford recommended medicines	Identify and help patient sign up for prescription assistance programs; Interface with physician(s) to discuss less costly alternatives.
SUD treatment program requires photo ID	Identify nearest DMV; Assist with transportation (bus pass; cab fare); Assist with processing fee.
Recommended care is far or inconvenient	Assist with transportation (bus pass; cab fare); Facilitate transfer to more convenient providers.
Missed appointment	Reschedule; Remind patient about appointment; Accompany patient to appointment.

**Table 2.** Data collection schedule and measures.

Measures	Baseline	3-month	6-month	12-month
Hospitalizations and ED visits ^1^		♦	♦	♦
Index diagnoses	♦			
Illicit drug use (biological test)		♦	♦	♦
Substance use patterns (ASI-Lite)	♦	♦	♦	♦
SUD diagnostic criteria	♦	♦	♦	♦
HIV risk behavior (Risk Assessment Battery)	♦	♦	♦	♦
Quality of Life (WHOQOL-BREF)	♦	♦	♦	♦
Psychological distress (Kessler-6)	♦	♦	♦	♦
Patient satisfaction		♦		
Health service utilization (Economic Form 90)	♦	♦	♦	♦
Economic costs (Economic Form 90)	♦	♦	♦	♦

1 Continuous data abstraction from health information exchange and electronic health records.
